# Two-dimensional Co-based metal-organic framework nanosheets as an efficient electrochemical sensing platform for simultaneous determination of daunorubicin and idarubicin

**DOI:** 10.5599/admet.2686

**Published:** 2025-03-05

**Authors:** Somayeh Tajik, Erfan Beiromi, Hadi Beitollahi, Fariba Garkani Nejad, Zahra Dourandish

**Affiliations:** 1 Research Center of Tropical and Infectious Diseases, Kerman University of Medical Sciences, Kerman, Iran; 2 Faculty of Pharmacy, Kerman University of Medical Sciences, Kerman, Iran; 3 Environment Department, Institute of Science and High Technology and Environmental Sciences, Graduate University of Advanced Technology, Kerman, Iran

**Keywords:** Chemotherapy, 2D Co-MOF nanosheets, electrochemical sensing platform

## Abstract

**Background and purpose:**

Chemotherapy is the most effective and commonly utilized cancer treatment method. Therefore, studies on the sensitive determination of chemotherapy drugs used in cancer treatment can be very effective in improving treatment and reducing their side effects.

**Experimental approach:**

The two-dimensional Co-based metal-organic framework nanosheets (2D Co-MOF NSs) were synthesized and then utilized to modify the screen-printed carbon electrode (2D Co-MOF NSs/SPCE). The 2D Co-MOF NSs/SPCE was successfully used for the determination of daunorubicin (DNR). Furthermore, we utilized differential pulse voltammetry, cyclic voltammetry, and chronoamperometry to evaluate the electrochemical properties of the created electrode.

**Key results:**

The obtained results from CV studies demonstrate that this sensor exhibits outstanding electrocatalytic activity for the redox process of DNR. Under optimal experimental conditions, quantitative measurements resulted in a linear concentration range from 0.004 to 450.0 μM for DNR with a limit of detection (LOD) of 0.001 μM. Furthermore, the fabricated electrode was used for the simultaneous voltammetric detection of DNR and idarubicin (IDR). According to the results, the 2D Co-MOF NSs/SPCE sensor showed two well-defined peaks for the voltammetric oxidation of DNR and IDR. Eventually, the practical sample detection of DNR and IDR was successfully validated with acceptable results.

**Conclusion:**

The developed sensing platform will be beneficial for enabling effective medical strategies to improve the clinical efficacy of chemotherapy drugs.

## Introduction

Cancer is one of the most severe, fatal, and frequently occurring diseases worldwide. Cancer originates when cells within the body grow uncontrollably [1,2]. Acute leukaemia, also known as blood cancer, is a prevalent life-threatening condition that typically initiates in the bone marrow, leading to an excess of immature white blood cells. The deoxyribonucleic acid (DNA) of these cells is either damaged or altered to prevent them from dying when they should, causing them to accumulate and occupy more and more space. Eventually, this leads to bone marrow failure [3,4]. Blood cancers are often challenging to cure entirely and frequently present with a poor prognosis. The chemotherapy drugs used target the cell division process in cancer cells. Among the current drugs, idarubicin (IDR) and daunorubicin (DNR) hold an outstanding position in the treatment of blood cancer [5]. DNR and IDR belong to the anthracycline antibiotics. The precise mechanism of DNR and IDR involves binding to DNA and inhibiting the production of nucleic acid by disrupting the molecular structure and creating a space barrier. Consequently, these drugs inhibit topoisomerase II activity within the cell [6-8]. Nonetheless, these drugs can cause various side effects, including anaemia, nausea, hair loss, vomiting, allergic reactions, tinnitus, diarrhoea, fertility issues, fever, bronchospasm, flu-like symptoms, and an increased risk of secondary malignancies [9,10]. Consequently, the determination of DNR and IDR levels is crucial from a clinical perspective. There are some analytical techniques, such as high-performance liquid chromatography (HPLC) [11], fluorescence spectrometry [12], and capillary electrophoresis [13,14], which can be applied to determine these drugs. While reliable and accurate, these techniques can be costly and time-consuming, encouraging researchers to create new, more efficient approaches. In recent years, electrochemical sensing has attracted significant interest in electroactive species due to its low cost, simplicity, and portability [15-18]. These techniques offer the advantage of relatively short analysis times. Among different analytical techniques, electrochemical techniques are relatively accurate and sensitive, making them ideal for applications in agriculture, food, and medicine [19-21]. Developing an appropriate platform in the electrochemical approach is of great importance.

Lately, screen-printed electrodes (SPEs) have attracted significant interest due to their ability to challenge the conventional three-electrode cell system [22]. This is primarily because of their low cost, simplicity, portability, and large-scale production. These appealing features facilitate the transition of electrochemical laboratory investigations to on-site determination of diverse analytes. In particular, recent investigations have demonstrated that the modification process of the surface of electrodes using different strategies can be significantly effective in the development of electrochemical sensors [23,24]. Various chemically modified electrodes for electroanalysis and application in diverse sensors have been devised [25,26]. Compared to un-modified electrodes, modified electrodes enhance the electrochemical response and sensitivity while lowering the limit of detection and reducing electrode fouling [27]. In this context, modification of the electrode surface by utilizing various effective materials is highly encouraged. Recent studies revealed that using nanomaterials in electrode modifications can revolutionize the field of electrochemical sensing by enabling rapid and sensitive detection of different substances with potential applications in healthcare, environmental monitoring, and food safety [28-31].

Metal-organic frameworks (MOFs) refer to porous materials originating from zeolite chemistry and coordination chemistry. MOF is a porous crystalline structure composed of metal ions connected by organic ligands. The properties and structure of MOFs can be continuously tailored according to the research and function by adjusting the metal ions and ligands [32,33]. Two-dimensional (2D) MOFs have been extensively utilized in sensing technology, electrochemical energy storage, and catalytic reactions thanks to their exceptional ion diffusion, large specific surface area, and electron-rich active centre [34,35]. The utilization of 2D cobalt-based MOF nanomaterials (2D Co-MOF NSs) as an electrode material for electrochemical sensors has emerged as a topic of significant research interest.

In this study, 2D Co-MOF NSs were synthesized and characterized using X-ray diffraction (XRD) and field-emission scanning electron microscopy (FE-SEM). The synthesized 2D Co-MOF NSs material was subsequently drop-cast onto a screen-printed carbon electrode (2D Co-MOF NSs/SPCE) to create a new electrochemical sensing platform for the detection of DNR. Due to its customized surface properties, 2D Co-MOF NSs exhibited significantly improved electrochemical activity for the redox process of DNR. Also, the 2D Co-MOF NSs modified SPCE was applied for the simultaneous determination of DNR and IDR. The proposed sensor showed two distinct oxidation peaks for these species. Additionally, the developed sensing platform was effectively used to detect the levels of DNR and IDR in real samples, demonstrating high accuracy and practicality.

## Experimental

### Chemicals and instrumentations

Daunorubicin, idarubicin, 2-aminoterephthalic acid, Co(NO_3_)_2_×6H_2_O, and other chemicals were obtained from Merck and Sigma-Aldrich. The used reagents were of analytical quality and without additional preparations and purifications. The buffer solution (phosphate buffer solution (PBS)-0.1 M), as the supporting electrolyte solution, was prepared by using phosphoric acid (H_3_PO_4_). The solutions were adjusted at the desired pH value with sodium hydroxide (NaOH) solution using a pH meter (model 713-Metrohm- Switzerland). The electrochemical investigations and measurements, including voltammetric tests (CV and DPV) and chronoamperometric studies, were carried out using a PGSTAT 302N-electrochemical workstation (Metrohm, The Netherlands) connected to a computer. The screen-printed carbon electrodes (SPCEs) consist of a three-electrode configuration (working, reference, and counter electrodes). They have carbon working electrode (WE), carbon counter electrode (CE), and Ag-pseudo reference electrode (RE).

### Synthesis of 2D Co-MOF NSs

The preparation of 2D Co-MOF NSs was performed according to the method reported by Li *et al.* with some modifications [36]. At first, 0.86 mmol of Co(NO_3_)_2_×6H_2_O (0.25 g) was added to 20 mL of deionized water. Then, a separate solution was prepared by adding 0.4 mmol (0.072 g) of 2-aminobenzene-1,4-dicarboxylic acid (2**-**aminoterephthalic acid) and 0.25 g of polyvinylpyrrolidone (PVP) into a mixture of ethanol (20 mL) and N,N-dimethylformamide (DMF) (20 mL). This solution was stirred for about 30 min at ambient temperature. After that, the aqueous solution of Co(NO_3_)_2_ was added slowly to the above solution. After stirring for 30 min, they were mixed and transferred into a Teflon-lined autoclave. The hydro/solvothermal reaction of precursors was carried out by placing the autoclave into an oven to heat for 24 h at 80 °C. After completion of the reaction, the autoclave was removed from the oven and gradually cooled. The precipitation of prepared 2D Co-MOF NSs was separated from the solution through centrifugation at 6000 rpm for 7 min. Finally, the precipitate was washed with the mixture of deionized water and ethanol for several times and then dried at 65 °C for 15 h.

### Modification and preparation of working electrode

2D Co-MOF NSs-modified SPCE was created using a straightforward drop-casting technique. Initially, 2D Co-MOF NSs suspension (1 mg/mL in water) was sonicated for 30 minutes, following which 3.0 μL of the suspension was drop-casted on the WE of SPCE. After evaporating the suspension solvent on the WE, the modified SPCE was prepared.

## Results and discussion

### Characterization of 2D Co-MOF NSs

**Figure 1.**
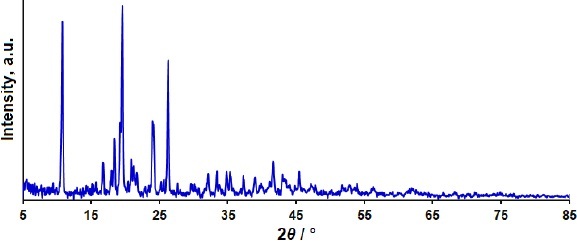
XRD pattern of 2D Co-MOF NSs

The crystalline structure of 2D Co-MOF NSs was evaluated by XRD (Figure 1). As observed from the XRD pattern, 2D Co-MOF NSs reveal characteristic peaks, which confirm their crystallinity. The observed XRD pattern corresponds to the obtained results in the previous report [36].

**Figure 2.**
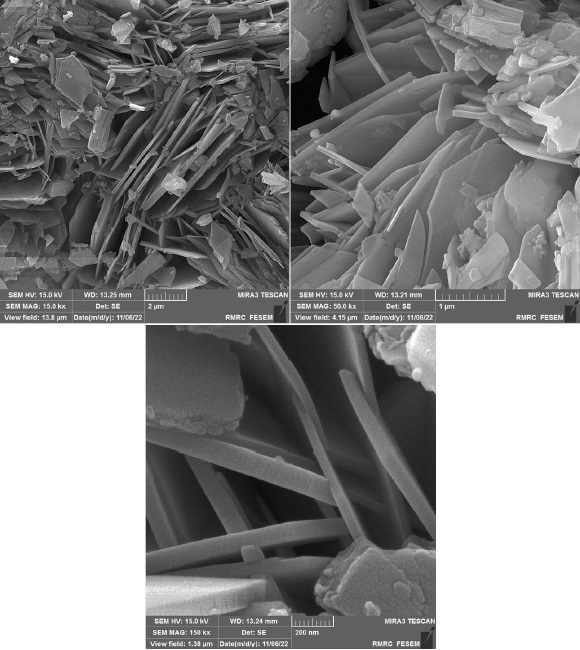
FE-SEM images of 2D Co-MOF NSs

Figure 2 depicts the FE-SEM images of as-prepared Co-based MOF at various magnifications. FE-SEM images reveal the formation of a two-dimensional sheet-like structure of Co-based MOF with a thickness of about below 100 nm.

### Electrochemical response of DNR at bare SPCE and 2D Co-MOF NSs/SPCE

The impact of pH on the voltammetric analysis of DNR was evaluated within the pH range of 2.0 to 9.0 using the DPV method. The findings indicated that as the pH increased, the anodic peak currents (*I*_pa_) of DNR also increased, and the highest *I*_pa_ of DNR was obtained at pH 7.0 and subsequently decreased. Hence, pH 7.0 was selected as the optimal pH value of PBS for further studies and measurements.

**Figure 3.**
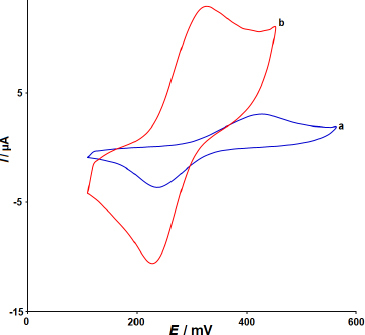
CVs of un-modified SPCE (a) and 2D Co-MOF NSs/SPCE (b) in pH 7.0 of 0.1 M PBS with 300.0 μM DNR at scan rate of 50 mV s^-1^

Figure 3 indicates the CV responses of un-modified SPCE (a) and 2D Co-MOF NSs/SPCE (b) in 0.1 M PBS with pH 7.0 containing 300.0 μM DNR at a scan rate of 50 mV s^−1^. In both voltammograms, the appearance of oxidation and reduction peaks revealed that the electrochemical process of DNR is redox under these conditions. The cyclic voltammogram a shows a weak redox peak for DNR on the surface of bare SPCE, demonstrating that the electron transfer occurs at a low rate on the surface of this electrode. After SPCE modification, the enhanced and well-defined redox peak currents (*I*_pa_ = 12.9 μA and *I*_pc_ = -10.6 μA) appear at considerably lower overpotentials 320 (*E*_pa_) and 230 mV (*E*_pc_) on the 2D Co-MOF NSs/SPCE. This facilitated electrochemical response towards DNR is associated with the high surface area of the developed electrode. Thus, 2D Co-MOF NSs can serve as an efficient material for the sensitive electrochemical detection of DNR.

### Investigation of scan rate at 2D Co-MOF NSs/SPCE

**Figure 4.**
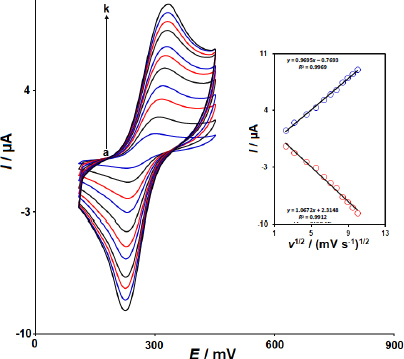
CVs of 150.0 μM DNR at the 2D Co-MOF NSs/SPCE in 0.1 M PBS (pH 7.0) at different scan rates, cyclic voltammograms a to k related to 5 (a), 10 (b), 20 (c), 30 (d), 40 (e), 50 (f), 60 (g), 70 (h), 80 (i), 90 (j) and 100 mV s^-1^ (k)

Figure 4 illustrates the CVs of 150.0 μM DNR in 0.1 M PBS (pH 7.0) on the 2D Co-MOF NSs/SPCE at various scan rates.

This experiment was conducted to study the kinetics of the electrode reactions and confirm whether diffusion solely dictates mass transport. The observation indicates that the redox peak currents (*I*_pa_ and *I*_pc_) gradually increase as the scan rate increases (Figure 4). As the scan rate increased, the redox potentials of DNR experienced a slight shift, suggesting kinetic constraints in the electrochemical reaction of DNR. Within the range of 5 to 100 mV s^-1^, the redox peak currents exhibited a proportional relationship to the square root of the scan rate (*v*^1/2^), suggesting that the electron transfer reaction was governed by a diffusion process.

### Chronoamperometric investigations

**Figure 5.**
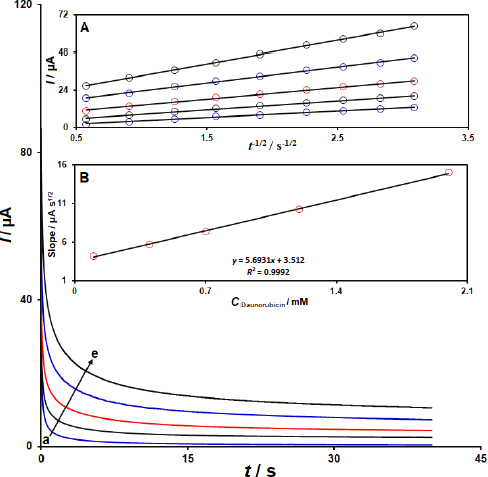
Chronoamperograms of 2D Co-MOF NSs/SPCE for DNR in 0.1 M PBS (pH 7.0); at a potential step of 370 mV for diverse concentrations of DNR (chronoamperograms related to C: 0.1 (a), C: 0.4 (b), C: 0.7 (c), C: 1.2 (d), and C: 2.0 mM (e) of DNR). Inset A: Plots of *I* vs. *t*^-1/2^ obtained from Cottrell's plot based on obtained chronoamperograms. Inset B: Plot of the slopes of the straight lines *vs.* the DNR concentrations

To calculate the diffusion coefficient, chronoamperograms for DNR were obtained using 2D Co-MOF NSs/SPCE at a constant potential of 370 mV (Figure 5). The DNR diffusion coefficient was determined using the Cottrell Equation (1):



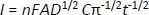

(1)


where *D* is the diffusion coefficient (cm^2^ s^-1^) and *C* is the analyte concentration (mol cm^-3^). The inset of (A) in Figure 5 exhibits the experimental plots of *I vs. t*^-1/2^ with the optimal fits for various concentrations of DNR used. Subsequently, the slopes of the obtained linear relationships were plotted against the DNR concentrations (inset (B) of Figure 5). By analyzing the regression slope and applying the Cottrell equation, the average *D* of 2.7×10^-6^ cm^2^ s^-1^ was calculated for DNR.

### Voltammetric detection of DNR on 2D Co-MOF NSs/SPCE

**Figure 6.**
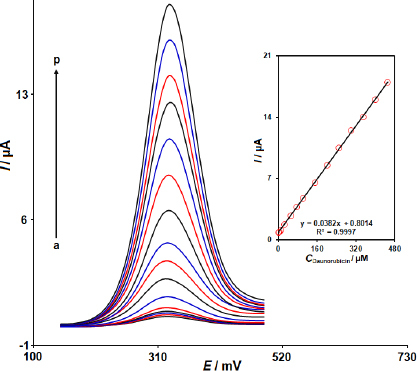
DPV responses at the 2D Co-MOF NSs/SPCE in 0.1 M PBS with pH 7.0 containing diverse concentrations of DNR (differential pulse voltammograms a to p related to 0.004, 0.05, 0.5, 2.5, 10.0, 25.0, 50.0, 75.0, 100.0, 150.0, 200.0, 250.0, 300.0, 350.0, 400.0, and 450.0 μM DNR, respectively). Inset: the plot of *I*_pa_ *vs.* the DNR concentration

Figure 6 illustrates the DPV responses at various concentrations of DNR on 2D Co-MOF NSs/SPCE and the related calibration curve. As depicted in Figure 6, the current responses rise as the concentration of DNR increases. Under optimal conditions, linearity was established with the equation *I*_p_ (μA) = 0.0382*C*_DNR_ (μM) + 0.8014; (*R*^2^ = 0.9997). A good linearity is achieved (Inset of Figure 6) when plotting anodic peak currents (*I*_pa_) against DNR concentrations ranging from 0.004 to 450.0 μM with a LOD of 0.001 μM based on *S*/*N* = 3. The sensitivity of the method was 0.0382 μA μM^-1^.

### Simultaneous determination of DNR and IDR

**Figure 7.**
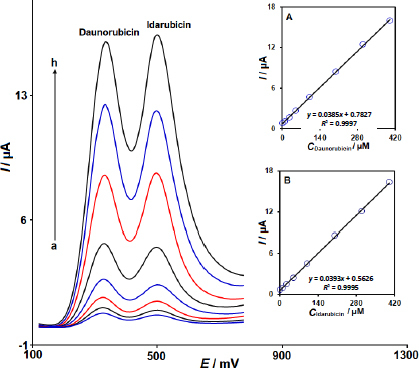
DPV responses at the 2D Co-MOF NSs/SPCE in 0.1 M PBS (pH 7.0) containing diverse concentrations of DNR and IDR (voltammograms a to h are corresponded to 0.5+0.5, 10.0+10.0, 25.0+25.0, 50.0+50.0, 100.0+100.0, 200.0+200.0, 300.0+300.0, and 400.0+400.0 μM of DNR and IDR, respectively). Insets: (A) the plot of *Ipa* vs. the DNR concentration and (B) the plot of *I*_pa_ *vs.* the IDR concentration

The DPVs of solutions with varying concentrations of DNR and IDR were obtained at 2D Co-MOF NSs/SPCE (Figure 7).

The DPVs depicting *I*_pa_ versus the concentration of DNR and IDR are displayed in Figures 7A and 7B, respectively. The slope observed in Figure 7A is close to that obtained in individual DNR analyses. Two distinct oxidation signals were detected in the solutions with varying concentrations of DNR and IDR with a separation of Δ*E* = 170 mV. These findings validate the performance of 2D Co-MOF NSs/SPCE as a voltammetric sensor for quantifying DNR in the presence of IDR.

### Practical application 2D Co-MOF NSs/SPCE sensor in real specimens

The suitability of 2D Co-MOF NSs/SPCE was assessed for the analysis of DNR and IDR in real specimens. These drugs were analysed in injection samples. The quantification of DNR and IDR in these samples was carried out using the standard addition approach. The outcomes are displayed in Table 1. The recoveries were also calculated, and the results are satisfactory, indicating that the proposed technique is effective for quantifying DNR and IDR trace levels in injection samples.

**Table 1.** Determination of DNR and IDR in real specimens using 2D Co-MOF NSs/SPCE sensor (*n* = 5).

Sample	Spiked concentration, μM		Found concentration, μM		Recovery, %		RSD, %	
DNR injection	DNR	IDR	DNR	IDR	DNR	IDR	DNR	IDR
	0	0	3.1	-	-	-	3.3	-
	1.0	5.0	4.0	5.1	97.6	102.0	2.9	2.3
	3.0	7.0	6.3	6.9	103.3	98.6	2.1	3.0
	5.0	9.0	8.2	8.9	101.2	98.9	1.7	2.0
	8.0	11.0	10.8	11.5	97.3	104.5	2.6	2.7
IDR injection	0	0	-	2.1	-	-	-	2.4
	5.5	3.0	5.6	5.0	101.8	98.0	3.1	2.8
	7.5	5.0	7.3	7.3	97.3	102.8	2.1	1.9
	9.5	7.0	9.8	9.0	103.2	98.9	1.8	2.2
	11.5	9.0	11.4	11.5	99.1	103.6	2.5	2.3

## Conclusion

In summary, the SPCE was modified with 2D Co-MOF NSs and employed as a voltammetric sensor for DNR detection. The electrochemical investigations show that the 2D Co-MOF NSs/SPCE sensor can enhance the voltammetric sensing of DNR compared with un-modified SPCE. Quantitative analysis was conducted using DPV, revealing a linear relationship between peak currents and DNR concentrations in the 0.004 to 450.0 μM range, with a LOD of 0.001 μM. In addition, the proposed sensor displayed good catalytic activity for highly sensitive detection of DNR in the presence of IDR. Finally, the 2D Co-MOF NSs/SPCE sensor was successfully used in the quantification of DNR and IDR in real specimens.
